# High Relaxivity with No Coordinated Waters: A Seemingly Paradoxical Behavior of [Gd(DOTP)]^5–^ Embedded in Nanogels

**DOI:** 10.1021/acs.inorgchem.2c00225

**Published:** 2022-03-22

**Authors:** Fabio Carniato, Marco Ricci, Lorenzo Tei, Francesca Garello, Enzo Terreno, Enrico Ravera, Giacomo Parigi, Claudio Luchinat, Mauro Botta

**Affiliations:** † Dipartimento di Scienze e Innovazione Tecnologica, Università del Piemonte Orientale “A. Avogadro”, Viale Teresa Michel 11, Alessandria 15121, Italy; ‡ Molecular Imaging Centre, Department of Molecular Biotechnology and Health Sciences, University of Torino, Via Nizza 52, Torino 10126, Italy; § Magnetic Resonance Center (CERM), University of Florence, via Sacconi 6, Sesto Fiorentino 50019, Italy; ∥ Department of Chemistry “Ugo Schiff”, University of Florence, via della Lastruccia 3, Sesto Fiorentino 50019, Italy; ⊥ Consorzio Interuniversitario Risonanze Magnetiche Metallo Proteine (CIRMMP), via Sacconi 6, Sesto Fiorentino 50019, Italy

## Abstract

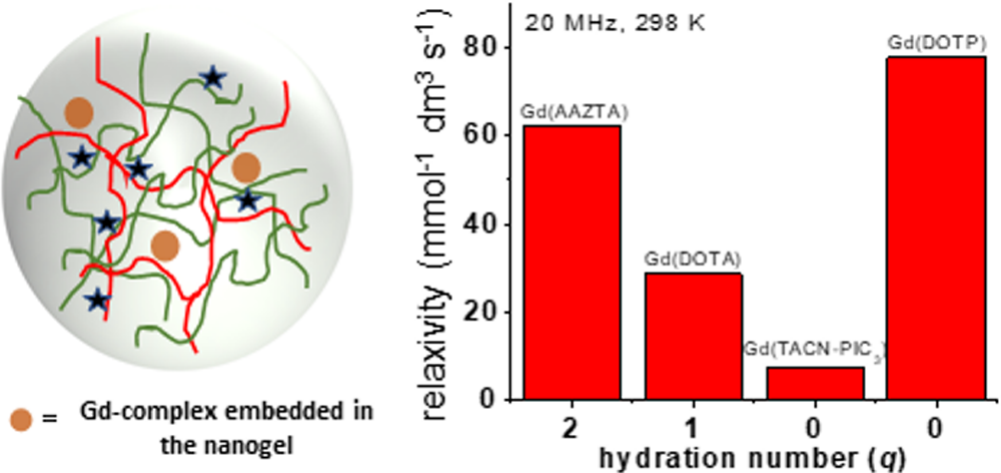

Nanogels (NGs) obtained by electrostatic interactions between chitosan and hyaluronic acid and comprising paramagnetic Gd chelates are gaining increasing attention for their potential application in magnetic resonance bioimaging. Herein, the macrocyclic complexes [Gd(DOTP)]^5−^, lacking metal-bound water molecules (*q* = 0), were confined or used as a cross-linker in this type of NG. Unlike the typical behavior of Gd complexes with *q* = 0, a remarkable relaxivity value of 78.0 mM^–1^ s^–1^ was measured at 20 MHz and 298 K, nearly 20 times greater than that found for the free complex. A careful analysis of the relaxation data emphasizes the fundamental role of second sphere water molecules with strong and long-lived hydrogen bonding interactions with the complex. Finally, PEGylated derivatives of nanoparticles were used for the first *in vivo* magnetic resonance imaging study of this type of NG, revealing a fast renal excretion of paramagnetic complexes after their release from the NGs.

## Abstract 2

Nanogels incorporating [Gd(DOTP)]^5−^ complexes (*q* = 0) exhibit remarkable relaxivity values, thanks to structured water molecules in the second coordination shell of the metal ion involved in strong H-bonding interactions with the phosphonate groups.

## Introduction

There is a growing interest in the study of nanogels (NGs) incorporating complexes of paramagnetic metal ions for applications in biomedicine and, in particular, in bioimaging.^1−5^ A number of properties particularly favorable for use in magnetic resonance imaging (MRI) preclinical applications and for a possible clinical translation stimulate the interest in these nanoprobes. For instance, characteristic properties, such as the size, shape, charge, degradability, and biocompatibility, can be optimized and controlled through an appropriate choice of the chemical composition.^6,7^ The nanoparticles can be loaded with a broad variety of compounds, and this allows their flexible use for a wide range of applications, especially biomedical imaging.^8^ Finally, appropriately sized NGs (approx. <200 nm) are able to avoid uptake by the reticuloendothelial system, which allows for longer circulation times and increased imaging windows.

The metal complexes that are used in clinical MRI are based on Gd^3+^.^9−11^ Gd-based contrast agents (GBCAs) have been investigated for over 3 decades and therefore we have gained a thorough understanding of their magnetic properties and structure–property relationships. Their effectiveness as *T*_1_ shortening agents is measured by a parameter called relaxivity (*r*_1_, mM^–1^ s^–1^), which corresponds to the relaxation rate enhancement of water protons normalized to 1 mM concentration of the paramagnetic metal ion. Molecular tumbling, described in terms of the rotational correlation time τ_R_, predominantly controls relaxivity. Because of their low molecular weight (low values of τ_R_), the commercially available GBCAs have modest values of *r*_1_ and, consequently, a relatively poor efficacy in clinical magnetic fields (1.5–3 T).

In preclinical applications, the inherent low sensitivity of MRI plays an important role, as the lowest Gd concentration detectable falls in the micromolar range; hence, signal amplification strategies are essential. It is well recognized that the use of nanosized systems decorated with a high number of Gd^3+^ complexes (10^3^ to 10^5^ complexes per particle) represents an effective strategy to overcome this limitation.^12^ In fact, not only does each nanoparticle carry a very high number of paramagnetic ions but also, due to their drastically reduced rotational dynamics (long τ_R_ values), the relaxivity of each of the conjugated Gd complex is markedly increased compared to that of the free complex (short τ_R_). Over the last 15 years, a large number of paramagnetic nanosystems composed of functionalized gold-, silica-, and lipid-based nanoparticles, and supramolecular adducts have shown enhanced relaxivity values per metal ion and per particle.^12−21^

Concerns on GBCAs (especially acyclic complexes) were recently raised due to gadolinium accumulation in tissues of patients who have been administered multiple doses during MRI examinations.^22,23^ This stimulated the search for possible alternatives (Mn- or Fe-based CAs) or imaging probes of enhanced safety.^24−30^ A possible strategy could involve the use of NGs incorporating suitable Gd^3+^ chelates. In fact, NGs loaded with small Gd^3+^ chelates could reduce their availability to unwanted biotransformation, as the complexes would be protected from transmetallation reactions, one of the mechanisms underlying the deposition of Gd^3+^ from acyclic complexes observed *in vivo*.

Recently, NGs obtained by electrostatic interactions between biocompatible chitosan and hyaluronic acid (NGs) have been synthesized incorporating Gd^3+^ complexes with different coordination geometries and hydration states: the clinically approved [Gd(DOTA)(H_2_O)]^−^ and the bis-hydrated [Gd(AAZTA)(H_2_O)_2_]^−^. The final NGs showed high *r*_1_ values at clinical fields due to the presence of an inner sphere water molecule(s) in fast exchange with the bulk and to a constrained mobility of the chelates included in the NGs.^4^

A few years ago, similar NGs were reported encapsulating the macrocyclic complexes [Gd(DOTP)]^5–^, which do not have any metal-bound water molecule (*q* = 0). For such nanoparticles, values of *r*_1_ up to approx. 100 mM^–1^ s^–1^ at 30 MHz were measured, which nearly correspond to the maximum values predicted by the theory for monohydrate Gd complexes.^1^ These experimental observations, mainly of qualitative nature, have not yet been analyzed in terms of a relaxation model. The isolated [Gd(DOTP)]^5–^ complex itself is known to exhibit surprising and unusual properties. About 2 decades ago, it was observed that its relaxivity, over a wide range of frequencies, reproduced that of corresponding monohydrate complexes despite the absence of a metal-bound water molecule.^31,32^ This was accounted for by the presence of structured solvent molecules in the second coordination shell of the metal ion involved in relatively strong H-bonding interactions with the phosphonate groups.^31,32^ We propose, as an initial hypothesis, that the presence of an array of water molecules involved in long-life hydrogen bonding interactions with polar groups of the complex and arranged in the tightly confined environment of NGs may significantly influence the observed relaxivity.

**Figure 1:**
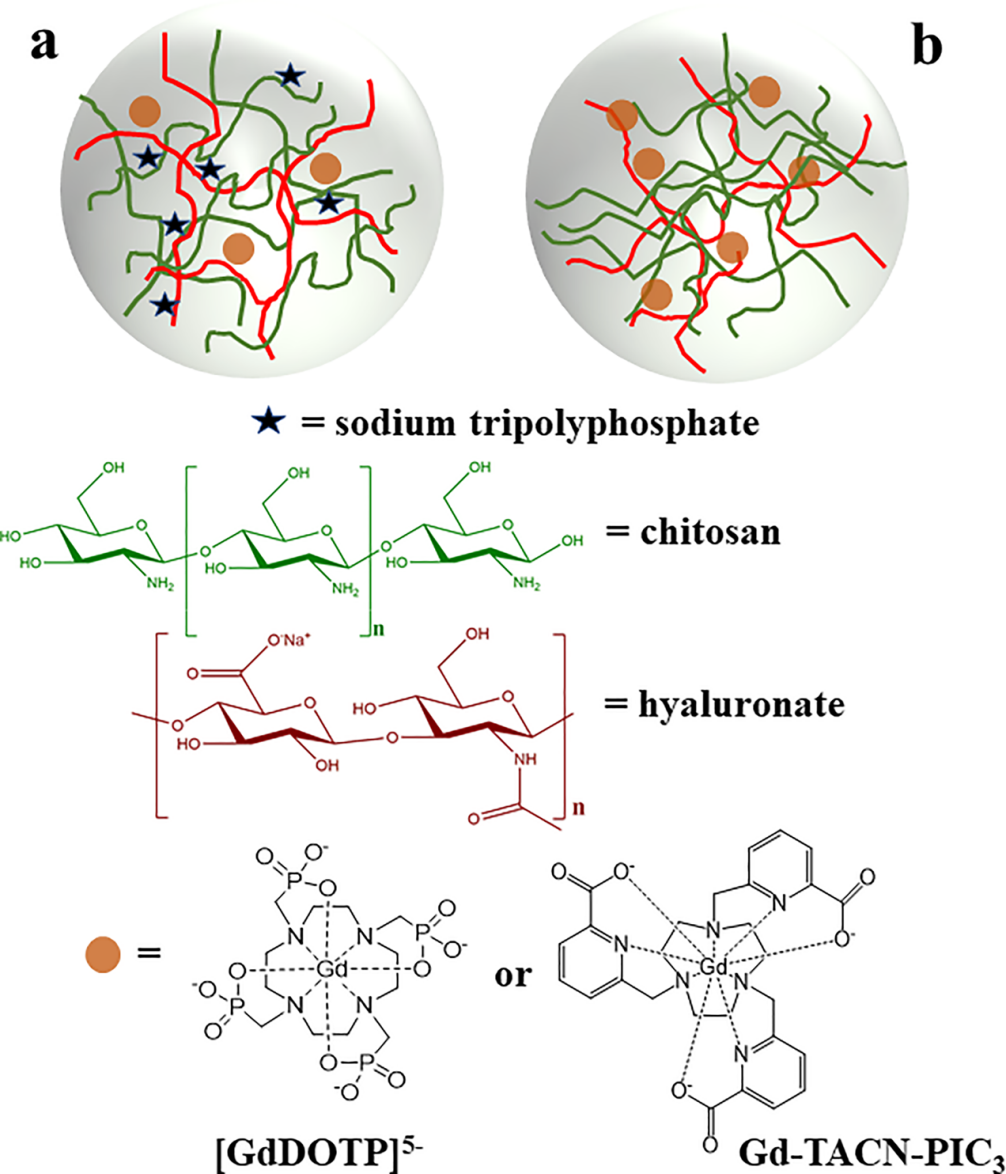
Schematic view of NG-1 (a) and NG-2 (b) formulations.

In this work, [Gd(DOTP)]^5–^ was confined in a NG matrix based on chitosan and hyaluronic acid, following two different synthetic strategies (NG-1 and NG-2), described in detail in the Materials and Methods section (Figure 1). For comparison, we also prepared homologous nanoparticles (NG-3) containing a *q* = 0 Gd^3+^ complex of similar size but not characterized by any contribution associated with water molecules of the second coordination sphere. This complex is based on a triazacyclononane ring with three picolinate pendant arms (Gd–TACN–PIC_3_, Figure 1), and its relaxivity is only determined by the outer sphere (OS) contribution.^33^ Besides morphological characterization, the *r*_1_ values of different NGs were measured as a function of the applied magnetic field and temperature, and the data were analyzed for assessing the values of molecular parameters (structural, dynamic, and electronic) that determine the relaxivity enhancement.

## Materials and Methods

### Chemicals and Materials

All chemicals were purchased and used without further purification. Electrospray ionization (ESI) mass spectra were recorded using a SQD 3100 mass detector (Waters), operating in the positive or negative ion mode, with 1% v/v formic acid in methanol as the carrier solvent.

#### [GdDOTP]^5–^

100 mg (0.18 mmol) of 1,4,7,10-tetraazacyclododecane-1,4,7,10-tetra(methylene phosphonic acid) (DOTP) ligand was dissolved in 2 mL of ultrapure water, and the pH was corrected to 7.0 with 2 M NaOH. An equimolar amount of GdCl_3_·6H_2_O (66.9 mg, 0.18 mmol) was slowly added to the solution, and the reaction was carried out at 333 K and neutralized with 2 M NaOH. A white precipitate was obtained during the synthesis. The product was dissolved by increasing the pH to 9. Finally, a slight excess (∼1%) of the ligand was added to the solution in order to remove the possible traces of uncomplexed Gd^3+^. MS ESI^–^ (*m*/*z*) = 702.2 [M]^−^; calculated C_14_H_28_GdN_4_O_12_P_4_ = 701.4 g mol^–1^.

#### NG-1

The NG was prepared by modifying a procedure described in the literature.^1^ 44.7 mg of chitosan (Sigma-Aldrich 740063-5G, molecular weight: 60–120 kDa) was dissolved in 18 mL of acetic acid solution (10 wt %). In a second flask, 11.4 mg of sodium tripolyphosphate (TPP, Alfa Aesar 13440), 8.0 mg of sodium hyaluronate (Hya, Alfa Aesar J66993, molecular weight greater than 1 MDa), and 18 mg of [Gd(DOTP)]^5–^ were solubilized in 9.0 mL of ultrapure water. The concentration of [Gd(DOTP)]^5–^ was 0.9 mM. After 30 min, the second solution was added dropwise on the first, and the reaction was stirred for 30 min at 298 K, thus promoting the generation of dispersed nanoparticles. The gelation reaction was very fast and occurred in a few minutes at 298 K in an acidic solution (pH = 3.0). The white suspension was purified by dialysis using a membrane with a cutoff of 14 kDa in pure water, and the purification procedure was repeated for 3 days in order to remove the unreacted compounds. The pH of the NG suspensions changed from 3.0 to 5.4 during the dialysis purification.

#### NG-2

The synthesis of the second formulation was carried out in a similar way, but the 11.4 mg of TPP used as a cross-linking agent in NG-1 was completely replaced by the Gd^3+^ chelate. The total amount of [GdDOTP]^5–^ used in the reaction was 40 mg ([Gd^3+^] = 2.1 mM).

#### NG-3

The NG was prepared and purified following the same synthetic approach and the same amount of reactants used for the synthesis of NG-1. In this case, [Gd(DOTP)]^5–^ was replaced by a *q* = 0 Gd^3+^ chelate based on a triazacyclononane ring with three pyridyl-2-carboxylate pendant arms.^33^ The concentration of the chelate confined in the NG after purification was calculated to be 4.1%.

#### NG PEGylation

150 mg of poly(ethylene glycol) (PEG)–COOH (MM = 3000 g mol^–1^) was dissolved in 5 mL of water in the presence of *N*′-(3-dimethylaminopropyl)-*N*-ethylcarbodiimide (0.05 mmol) and hydroxy-2,5-dioxopyrrolidine-3-sulfonic acid sodium salt (sulfoNHS) (0.2 mmol). The activated PEG was added to 8 mL of NG-1 or NG-2 suspensions. The suspensions were stirred for 24 h at room temperature and purified by dialysis using a membrane with a cutoff of 14 kDa in pure water at neutral pH.

### Characterization Techniques

The elemental analyses were performed on a Thermo Fisher Scientific X5 series inductively coupled plasma mass spectrometer (Waltham, MA, USA). The NGs were dehydrated, and the final solids were mineralized with 10 mL of nitric acid at 373 K for 24 h.

The Gd^3+^ concentration in the aqueous suspension was determined by inductively coupled plasma mass spectrometry (ICP-MS) and ^1^H NMR measurements using the bulk magnetic susceptibility method with a Bruker Avance III spectrometer equipped with a wide bore 11.7 T magnet.^34^ The quantification of chelates per nanoparticle was carried out following the procedure reported in the literature for similar NGs.^4^

The water content in the NGs was quantified by a gravimetric approach. 5 mL of both the NG suspensions was centrifuged at 10,000 rpm for 1 h at 7 °C. The hydrated solid was separated by the liquid and weighted. Then, the powders were dehydrated at 100 °C for 24 h and weighted again. The amount of entrapped water was calculated by applying the following equation
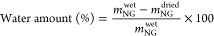
1

The water content in the two samples was found to be *ca.* 95%, in agreement with the data obtained for parent samples.^4^

Cryo-transmission electron microscopy (cryo-TEM) images were collected with a Thermo Scientific Glacios cryo-transmission electron microscope cryo-TEM equipped with a 200 kV X-FEG optics. The samples were prepared in a Vitrobot preparation chamber at ambient temperature. Small volumes of solution were deposited in a carbon-filmed grid and vitrified in liquid ethane at its freezing point (89 K).

Dynamic light scattering (DLS) and *Z*-potential experiments were carried out at 298 K by using a Malvern Zetasizer NanoZS operating in a particle size range from 0.6 nm to 6 mm and equipped with a He–Ne laser with λ = 633 nm.

1/*T*_1_^1^H nuclear magnetic relaxation dispersion (NMRD) profiles were measured on a fast-field cycling Stelar SmarTracer relaxometer over a continuum of magnetic field strengths from 0.00024 to 0.25 T (0.01–10 MHz proton Larmor frequencies). This relaxometer operates under computer control with an absolute uncertainty in 1/*T*_1_ of ±1%. Data in the 20–120 MHz frequency range were collected with a high-field relaxometer (Stelar) equipped with the HTS-110 3 T metrology cryogen-free superconducting magnet. The data were collected using the standard inversion recovery sequence with a typical 90° pulse width of 3.5 μs, and the reproducibility of the data was within ±0.5%.

The diamagnetic contribution was measured by collecting ^1^H NMRD profiles of the unloaded nanoparticles at different temperatures.

*In vitro* stability tests were carried out in water, human serum, or (4-(2-hydroxyethyl)-1-piperazineethanesulfonic acid) (HEPES)/NaCl buffer as reported in the Supporting Information.

### Calculation of *r*_1_ Values of NG-1 and NG-2

As previously described for NGs functionalized with mono- and bis-hydrated Gd^3+^ chelates, the relaxation rate of NG-1 and NG-2 suspensions can be defined by eq 2(4)
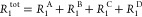
2in which *R*_1_^A^ is the relaxation contribution of the complexes entrapped inside the matrix, *R*_1_^B^ corresponds to the contribution of complexes weakly adsorbed on the external surface, and *R*_1_^C^ to that of free Gd^3+^ chelates. *R*_1_^D^ is the contribution of the diamagnetic nanoparticles (without complex). The terms *R*_1_^B^ and *R*_1_^C^ can be neglected for both NG-1 and NG-2 samples because the free complex was removed during the dialysis steps, and we have no evidence of the presence of chelates adsorbed on the external surface. Ultra-centrifugation tests applied for similar samples,^4^ extremely efficient for removing this weakly interacting fraction, support the conclusion that the relaxivity values measured for both NG-1 and NG-2 depend on the *R*_1_^A^ contribution, as indicated in eq 3
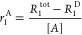
3

### *In Vivo* MRI Methods

The animal study was approved by the Italian Ministry of Health, and the followed procedures were in accordance with institutional guidelines and ensured the humane care of animals. The male BALB/c mice (aged 12 weeks and weighing 25–28 g) were obtained from the animal facility at the Molecular Biotechnology Center of the University of Turin. The animals received standard rodent chow and had free access to tap water.

For the MRI experiments, mice were anesthetized by the intramuscular injection of a mixture of tiletamine/zolazepam (Zoletil 100; Virbac, Milan, Italy) 20 mg/kg and xylazine (Rompun; Bayer, Milan, Italy) 5 mg/kg, catheterized, and placed supine in a solenoid Tx/Rx coil with an inner diameter of 3.5 cm. MRI was performed with an Aspect M2 MRI system (Aspect Magnet Technologies Ltd. Netanya, Israel) working at 1 T.

After the scout image acquisition, a *T*_2_-weighted anatomical image was acquired with a fast spin-echo sequence [repetition time (TR), 2500 ms; echo time (TE), 48 ms; number of averages (NAV), 4; number of slices, 4; slice thickness, 2 mm; slice gap, 0.1 mm; field of view (FOV), 40 mm, matrix, 128 × 128; and acquisition time, 2 min 40 s].

Dynamic contrast-enhanced MRI was performed using a *T*_1_-weighted gradient echo SNAP sequence (TR, 16 ms; TE, 2.7 ms; NAV, 5; number of repetitions, 40; number of slices, 4; slice thickness, 2 mm; slice gap, 0.1 mm; FOV, 40 mm; matrix, 128 × 128; acquisition time, 28 min; and flip angle, 30°). Three pre-contrast images were acquired, then the contrast agent was injected intravenously (6.5 μmol Gd/kg), and 37 post-contrast images were acquired.

To evaluate the % *T*_1_ signal enhancement, regions of interest were drawn around the kidneys, spleen, and liver, and the *T*_1_ signal intensity (SI) before and after the NG injection was assessed.

The % *T*_1_ signal enhancement over pre-images was then calculated as follows for each acquisition time in kidneys, liver, and spleen
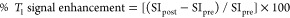
4where SI_pre_ is the average *T*_1_ SI of the three pre-contrast images in each organ, and SI_post_ is the *T*_1_ SI measured every 42 s post-injection for 37 timepoints.

## Results and Discussion

**Figure 2:**
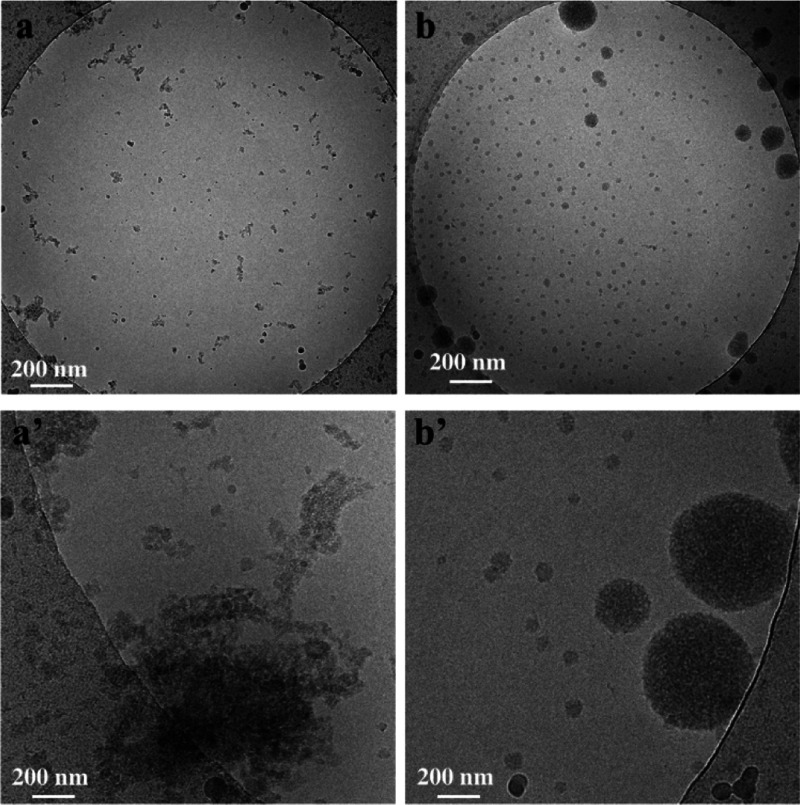
Cryo-EM images at low (a,b) and high magnifications (a′,b′) of NG-1 (a,a′) and NG-2 (b,b′).

The two different NG formulations were synthesized, adapting a literature procedure, by ionotropic gelation of the cationic chitosan polymer with sodium hyaluronate anionic compound. In the case of NG-1 (Figure 1a), TPP was chosen as an anionic cross-linker, and the NG preparation was carried out in the presence of a few mg of [Gd(DOTP)]^5–^ (Figure S1A and Scheme S1). However, through this approach, the Gd^3+^ loading was very limited and not sufficient for *in vivo* applications. To overcome this problem and increase the concentration of confined paramagnetic centers, in the second procedure (NG-2), [Gd(DOTP)]^5–^ was directly used as a cross-linking agent, in place of TPP (Figure S1B and Scheme S2). More experimental details are reported in the Materials and Methods section. The confinement of the Gd^3+^ chelates took place during the particles’ growth by exploiting multiple ionic interactions with the positive groups of chitosan. NG-1 and NG-2 formulations differ in the particle size, morphology, surface properties, Gd^3+^ loading, and relaxometric properties. To investigate the influence of the preparation procedure on the particle morphology, cryo-TEM images of NG-1 and NG-2 at low and high magnification were collected and compared. The NG-1 sample is composed of particles with sizes below 50 nm and irregular morphology, often experiencing mutual interactions that originate large aggregates of a few hundreds of nanometers (Figures 2 and S2). This is clearly visible in Figure 2a′, which shows islands composed of a number of nanosized particles of irregular shapes. Conversely, different morphological features characterize the NG-2 sample. No aggregates were detected at low and high magnifications (Figure 2b,b′).

The sample is composed of very small NPs with sizes below 50 nm, which characterize more than 70% of the sample and large spheres with diameter in the 70–180 nm range. The particles show a homogeneous shape with a spheroidal geometry and a fibrous network due to the overall hydration of the polymer chains. Similar behavior was also observed in aqueous solution by analyzing with DLS the dispersion degree at 298 K of the colloidal suspensions. The hydrodynamic diameter distribution for NG-1 is centered at *ca.* 75 nm with a visible component at *ca.* 500 nm attributed to the particle aggregates, according to cryo-EM results (Figure S3a).

The polydispersity index (PDI) was 0.50 because of the presence of these sub-micron aggregates, testifying a heterogeneous distribution of the nanoparticles. These aggregates are not detected in the DLS of NG-2 (Figure S3a) which presents a hydrodynamic diameter of *ca.* 120 nm and a PDI of 0.3, a value quite similar to the other NGs. The ζ-potentials were +35.2 and +21.4 mV for NG-1 and NG-2, respectively (298 K; pH 5.4). The differences probably depend on the different architectures of the polymeric matrices and on the [Gd(DOTP)]^5–^ loading, *ca.* 3 times higher for NG-2. The positive surface charge density of these NGs allows for high stability of the suspensions that was evaluated by monitoring the longitudinal relaxation rate (*R*_1_) values for several weeks at pH 5.5 (Figure S3b). For the NG-2 formulation, the *R*_1_ values measured (298 K and 32 MHz) remain substantially constant for 20 days, thus indicating the absence of sedimentation. On the other hand, a marked decrease of *R*_1_ is observed after 2 weeks for NG-1. This result can be accounted for by the precipitation of the larger aggregates present in the suspension.

The amount of [Gd(DOTP)]^5–^ confined in the two NGs after purification was calculated to be 3.1% and 5.1% (w/w), corresponding to Gd concentrations in the NG-1 and NG-2 suspensions of 0.18 (1.8 × 10^5^ Gd^3+^ chelates per particle, see the Supporting Information) and 0.60 mM (3.5 × 10^6^ Gd^3+^ complexes per particle), respectively. Then, the relaxometric properties of NG-1 and NG-2 were investigated and compared with those of free [Gd(DOTP)]^5–^ in solution. The longitudinal relaxivity (*r*_1_) was measured as a function of the applied magnetic field strength, over an extended range of values, and temperatures.^35,36^ The relaxivity arises from the time-modulation of the magnetic dipolar interaction between the water molecules and the paramagnetic ion that, for [Gd(DOTP)]^5–^, involves two mechanisms: (i) dipolar interaction with the water molecules hydrogen bonded to the phosphonate groups of the chelate (second sphere contribution; SS) and (ii) long-range interaction with the bulk water molecules diffusing next to the complex (OS contribution).^32^ By assigning to the OS contribution typical values of the Gd^3+^ complexes and assuming the presence of four water molecules belonging to the second coordination sphere, in agreement with the literature data,^37^ we obtain an excellent fit of the *r*_1_ profiles of the free [Gd(DOTP)]^5–^ on the basis of a Gd–H distance of 3.53 Å, an average life time (τ_M_) of 1 ns, and a τ_R_ of 40 ps. The values of *r*_1_ comprise between *ca.* 7 mM^–1^ s^–1^ at low fields and 4 mM^–1^ s^–1^ at high fields (Figure S4 and Table S1).

**Figure 3:**
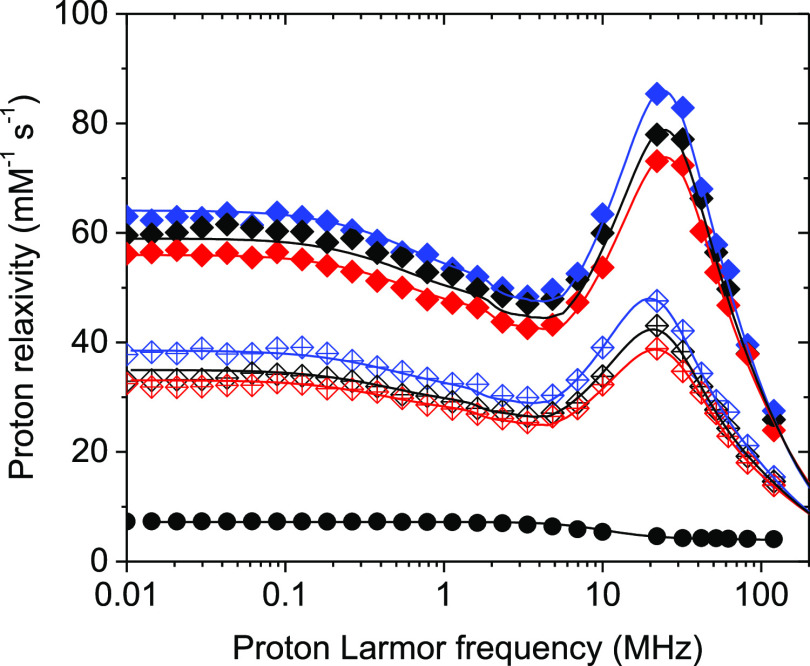
^1^H NMRD profiles at 283 (blue), 298 (black) and 310 K (red) of NG-1 ([Gd^3+^] = 0.18 mM) (⧫) and NG-2 ([Gd^3+^] = 0.60 mM) (◊) aqueous suspensions. The ^1^H NMRD profile of [Gd(DOTP)]^5–^ at 298 K is also reported (●).

The *r*_1_ values of NG-1 and NG-2 (Figure 3), measured at 283, 298 and 310 K, show a dramatic increase with respect to [Gd(DOTP)]^5–^ over the entire range of magnetic fields explored (0.01–120 MHz) (Table 1). In particular, NG-1 features a *r*_1_ maximum of 78.0 mM^–1^ s^–1^ at 20 MHz and 298 K, almost 20 times higher than that measured for the free complex under identical experimental conditions. In the case of NG-2, the increase is smaller, yet the *r*_1_ values observed are about 1 order of magnitude larger than those of the free Gd-chelate. It is worth noting that the relaxivity of NG-1 and NG-2 are much greater than those measured for [Gd(DOTP)]^5–^ in the presence of the individual constituents of the NG (TPP, chitosan, hyaluronate; Figure S5).

**Table 1:** *r*_1_ Values at 20, 60, and 120 MHz (298 K)

	^20^*r*_1_ (mM^–1^ s^–1^)	^60^*r*_1_ (mM^–1^ s^–1^)	^120^*r*_1_ (mM^–1^ s^–1^)
[Gd(DOTP)]^5–^	4.6	4.3	4.2
NG-1	77.9	49.7	25.9
NG-2	43.1	24.3	14.6

As mentioned above, similar chitosan-based NGs incorporating [Gd(DOTP)]^5–^ were reported previously, but the *r*_1_ values obtained were different.^1^ This is probably a consequence of the different properties of the NGs in terms of particle size, Gd^3+^ loading, preparation pathway, and molecular weight of chitosan. The most surprising and relevant result is that the proton relaxivities of NG-1 are appreciably higher than those measured for mono- or bis-hydrated complexes embedded in the same nanoparticle (Figure S6).^4^

The *r*_1_ profiles of NG-1 and NG-2 could be optimally fitted assuming for the parameters determining the OS contribution (distance of closest approach, *a*, and diffusion coefficient, *D*) the values that are typically found in Gd complexes. Particles of this size have an overall reorientation time (τ_R_) of at least hundreds of nanoseconds. The correlation time for nuclear relaxation is therefore independent of τ_R_. From the temperature dependence of the NMRD profiles, SS water molecules are in fast exchange with bulk solvent molecules. Under these conditions, assuming a proton–gadolinium(III) distance of 3.5 Å, 3.1 and 1.7 SS water molecules are found for NG-1 and NG-2, respectively, with a τ_M_ of *ca.* 6 ns. The variable number of second sphere water molecules in close proximity to [Gd(DOTP)]^5–^, in the two NGs, can justify the different *r*_1_ values of NG-1 and NG-2. Probably, the higher amount of SS water molecules in NG-1 can be attributed to the presence of TPP as a cross-linker that enables [Gd(DOTP)]^5–^ to maintain an almost unaltered second sphere of hydration (Figure S1). Furthermore, we can also hypothesize that the presence of TPP during the synthesis promotes the organization of a vast network of water molecules within the matrix through hydrogen bonding interactions, increasing their number in the vicinity of the metal complexes.

In the fit, a reorientation mobility of the SS water molecules inside the NG was also considered with a Lipari–Szabo^38^ order parameter *S*_LS_^2^ of *ca.* 0.35 and correlation times (τ_f_) of *ca.* 1 ns (see the Supporting Information). The fit of the low-field regions of the profiles also required considering the effect of static ZFS together in the presence of transient zero-field splitting (ZFS) (Δ_t_).^39−41^ All the best fit parameters are reported in Table S2. A τ_M_ value of some nanoseconds is optimal for achieving the largest relaxivities at magnetic fields of 1–1.5 T and provides relaxivity peaks close to the maximum values theoretically achievable (see Figure S7).

Therefore, the fast diffusion of the water molecules into the NG matrix and the exchange of the water molecules confined in NG-1 and NG-2 samples with the bulk water is sufficiently fast not to limit the relaxivity.

To emphasize the unusual behavior of [Gd(DOTP)]^5–^ and the relevant SS contribution to *r*_1_ for both NG-1 and NG-2, we prepared a novel NG with a chemical composition similar to NG-1 but functionalized with another *q* = 0 Gd chelate based on a triazacyclononane ring with three picolinate pendant arms (Gd–TACN–PIC_3_, NG-3), whose relaxivity is only determined by the OS contribution.^33^ Its relaxivity values at 298 K are markedly lower than those of NG-1 over the entire range of magnetic fields (*r*_1_ at 20 MHz = 7.6 mM^–1^ s^–1^; Figure S8).

*In vivo* MRI data of paramagnetic NGs are very rare;^42−44^ hence, we deemed of great interest testing our NGs on healthy mice. This study has the dual purpose of verifying the stability and the imaging performance of the nanoparticles under physiological conditions. Unfortunately, the NGs’ suspension displayed limited stability at physiological pH, forming aggregates. With the aim of improving stability, the nanoparticles were decorated on the surface with polyethylene glycol moieties^45^ by amide coupling between free NH_2_ groups on chitosan and PEG–COOH (MM = 3000 g mol^–1^), previously activated with *N*-hydroxysuccinimide. The final suspensions were purified by dialysis to eliminate the unreacted PEG molecules. The partial PEGylation of chitosan induces a slight increase of the hydrodynamic radius of both NG-1 and NG-2 (of *ca.* 30 nm, Figure S9). Importantly, the PEGylation caused a significant improvement in the stability of the NGs: no visible aggregation and no changes in the *R*_1_ values over time were observed at physiological pH both in water and in human serum (Figure S10). The presence on the surface of PEG moieties did not alter the relaxometric properties of the NG-1 sample, being mainly influenced by the local reorientation correlation time of the confined complex and by the second sphere hydration state of the metal, and marginally limits the relaxivity values of the NG-2 with a decrease <25% of *r*_1_ at 1 T (Supporting Information, Figure S11).

**Figure 4:**
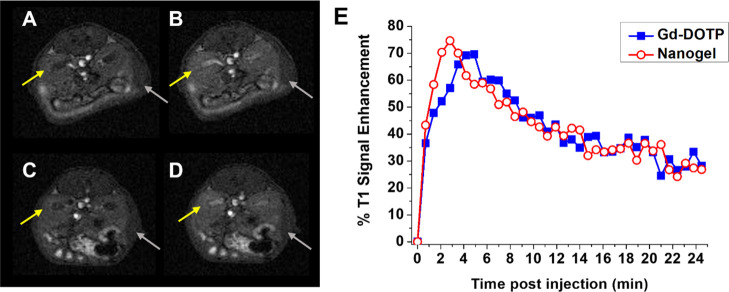
(A–D) MR images of kidneys (yellow arrows) and spleen (grey arrows) acquired at 1 T pre (A,C) and 3 min post-administration of (B) paramagnetic NG or (D) [Gd(DOTP)]^5–^. (E) % *T*_1_ signal enhancement over pre images measured in the kidney medulla after the administration of the paramagnetic NG (empty circles) or [Gd(DOTP)]^5–^ (filled squares) (6.5 μmol Gd/kg bw).

The MRI study was carried out at 1 T on healthy BALB/c mice, and only the PEGylated NG-2 sample was administered due to the larger amount of Gd^3+^ loaded as compared to NG-1. After the intravenous administration of NG-2–PEG (6.5 μmol Gd/kg), a marked *T*_1_ signal enhancement (*ca.* 75% over pre-contrast images) was measured into the kidneys, 3 min post-injection (Figure 4A,B,E), whereas no significant signal enhancement was detected in the other organs (Figure S12). The data obtained suggest a fast renal excretion of the paramagnetic complexes embedded into the NG.^46^ To verify this hypothesis, [Gd(DOTP)]^5–^ was administered intravenously in the absence of the NG (6.5 μmol Gd/kg) and the dynamic MRI acquisition was performed following the same protocol used for NG-2–PEG. The *T*_1_ signal enhancement profiles measured in the kidneys, liver, and spleen were comparable to the ones observed after the administration of the NG (Figures 4C–E and S12), further indicating the release and subsequent elimination of [Gd(DOTP)]^5–^ from the NG. This hypothesis was further confirmed *in vitro*, by performing sequential dialysis cycles on NG-2–PEG suspended in HEPES/NaCl buffer or in the human serum (Figure S13). In solutions of high ionic strength, the metal chelates are completely released from the NGs within 24 h.

## Conclusions

In conclusion, NGs incorporating [Gd(DOTP)]^5–^ complexes exhibit a surprising behavior that does not follow the typical paradigm.^46^ The absence of metal-bound water molecules does not markedly limit the efficiency of the complexes as relaxation agents but instead results in strongly enhanced values of relaxivity. A careful analysis of the relaxation data highlighted the fundamental role of SS water molecules. Three pools of exchanging protons that contribute to the network of SS relaxation can be envisaged for these systems: (i) water molecules in the bulk phase of the NG with sufficiently strong and long-lived hydrogen bonding interactions with the complex; (ii) exchangeable protons (mainly −OH and −NH_2_ groups) belonging to the polymeric backbone; and (iii) polymer hydration water molecules in rapid exchange with the water inside the nanoparticles. Although all three mechanisms can, in principle, contribute to the overall relaxivity, the high negative charge of the complex suggests that mechanism (i) could be the most relevant. This conclusion is also supported by the observation that the neutral Gd–TACN–PIC_3_ complex embedded in the same NG provides very low relaxivity. Finally, the first *in vivo* MRI study of chitosan/hyaluronic acid-based NGs incorporating Gd^3+^ complexes here reported revealed insufficient stability of the NG formulations post-administration. Therefore, future research efforts toward the development of paramagnetic NG-based probes should focus on optimizing the formulation strategies. The important goal is to significantly strengthen the interaction between the MRI agent and the NG components, while leaving unaltered, or possibly improve, the relaxation properties.

## Supporting Information Available

Synthesis procedures and characterization techniques, *in vivo* MRI experiments, schematic view of the synthesis of NG-1 and NG-2, schematic representation of the H-bonding network in NG-1 and NG-2, particle size distribution for NG-1 and NG-2, DLS analysis of the suspensions, relaxivity of [Gd(DOTP)]^5–^, ^1^H NMRD profiles of [Gd(DOTP)]^5–^ and the NGs, simulations of the ^1^H NMRD profiles of NG-1 and NG-2 samples with different τ_M_ values, parameters obtained by analysis of the NMRD profiles, DLS and NMRD profiles of pegylated NGs, stability of NG2–PEG in human serum at different times postincubation and after different dialysis times, and % of *T*_1_ signal enhancement over pre-images in the liver and spleen after administration of paramagnetic NG or [GdDOTP]^5–^ (PDF)
