# Supplementary material for: High Relaxivity with No Coordinated Waters: A Seemingly Paradoxical Behavior of [Gd(DOTP)]5– Embedded in Nanogels
Source: Inorg Chem. 2022 Mar 22;61(13):5380–7. doi: 10.1021/acs.inorgchem.2c00225 (PMC8985129; doi:10.1021/acs.inorgchem.2c00225)
Supplement: Supplementary file 1 — ic2c00225_si_001.pdf [file ic2c00225_si_001.pdf]

Supporting Information for

High relaxivity with no coordinated waters: a seemingly paradoxical behavior of $[Gd(DOTP)]^{5-}$ embedded into nanogels

Fabio Carniato,[a] Marco Ricci,[a] Lorenzo Tei,[a] Francesca Garello,[b] Enzo Terreno,[b]* Enrico Ravera,[c] Giacomo Parigi,[c] Claudio Luchinat,[c]* Mauro Botta[a]*

[a]*Dipartimento di Scienze e Innovazione Tecnologica, Università del Piemonte Orientale "A. Avogadro", Viale Teresa Michel 11, 15121, Alessandria, Italy; E-mail: mauro.botta@uniupo.it*
[b]*Molecular Imaging Centre, Department of Molecular Biotechnology and Health Sciences, University of Torino- Via Nizza 52, 10126 Torino, Italy; E-mail: enzo.terreno@unito.it*
[c]*Magnetic Resonance Center (CERM), University of Florence, via Sacconi 6, Sesto Fiorentino, 50019 Italy; Department of Chemistry "Ugo Schiff", University of Florence, via della Lastruccia 3, Sesto Fiorentino, 50019 Italy; and Consorzio Interuniversitario Risonanze Magnetiche Metallo Proteine (CIRMMP), via Sacconi 6, Sesto Fiorentino, 50019 Italy; E-mail: luchinat@cerm.unifi.it*

Contents:

Quantification of the number of chelates per nanoparticle p. S2
Field dependence of water proton relaxivity p. S3
In vitro stability tests p. S5
Schemes p. S6
Tables pp. S9-S10
Figures pp. S7-S16

Quantification of the number of chelates per nanoparticle (Y_{Gd}) for NG-1

Y_{Gd} was calculated as follows:

m% Gd determined by ICP-MS = 3.1 %

NP volume:

$v_{NP} = 4/3 \times \pi \times r_H^3$ nm^3, with r_H: mean hydrodynamic radius.

$v_{NP} = 4/3 \times \pi \times (33)^3 = 150455$ nm^3

v_{NP} (nm^3) = 0.15 x 10^{-15} cm^3

NP mass (g):

$m_{NP} = v_{NP} \times \rho_{NP} \rightarrow$ mNP = 0.15 x 10^{-15} g

ρ_{NP} was considered to be 1.0 g cm^{-3} considering the large amount of water entrapped in the nanoparticle. This value leads therefore to an underestimated result.

Number of Gd chelates per nanoparticle:

YGd = (mNP × m% Gd / MwGd) × NAvogadro which finally gives:

YGd = (0.15 x 10^{-15} × 0.031 / 157.25) × 6.02 × 10^{23} = 1.8 x 10^5 centers

The same procedure was also adopted for NG-2 sample.

Field dependence of water proton relaxivity

The difference in the longitudinal relaxation rates of solvent ligand nuclei between a paramagnetic solution and a diamagnetic reference solution is

$$R_{1p} = f_{\mathrm{M}}(R_{1M}^{-1} + \tau_{\mathrm{M}})^{-1} + R_{1\mathrm{OS}} \tag{1}$$

where R_{1M} is the relaxation rate of the ligand nuclei bound to the paramagnetic metal ion, τ_{M} is their exchange time, f_{M} is the mole fraction of ligand nuclei bound to the metal and $R_{1\mathrm{OS}}$ is the outer-sphere relaxation rate. The relaxivity r_1 is defined as the paramagnetic enhancement of the solvent nuclear relaxation rates in the presence of 0.001 mol/dm^3 of paramagnetic metal ions in solution. Therefore, the relaxivity in a water solution is

$$r_1 = \frac{0.001q}{55.6}(R_{1M}^{-1} + \tau_{\mathrm{M}})^{-1} + r_{1\mathrm{OS}} \tag{2}$$

where q is the number of water molecules coordinated to each paramagnetic metal ion and $r_{1\mathrm{OS}}$ is equal to $R_{1\mathrm{OS}}$ divided by the millimolar concentration of the paramagnetic metal ion. When $\tau_{\mathrm{M}} < R_{1M}^{-1}$, the coordinated water molecule(s) are in the so-called fast exchange.

The paramagnetic relaxation rate R_{1M} due to the point dipole-point dipole interaction between the water proton magnetic moment and the unpaired electron(s) magnetic moment is described by the Solomon equation

$$R_{1M} = \frac{2}{15}\left(\frac{\mu_0}{4\pi}\right)^2 \frac{\gamma_I^2 g_e^2 \mu_B^2 S(S+1)}{r^6}\left(\frac{7\tau_c}{1+\omega_S^2\tau_c^2} + \frac{3\tau_c}{1+\omega_I^2\tau_c^2}\right) \tag{3}$$

where μ_0 is the permeability of the vacuum, γ_I is the proton magnetogyric ratio, μ_B is the electron Bohr magneton, S is the electron spin quantum number, r is the distance between paramagnetic metal and proton of the coordinated water molecule, ω_S and ω_I are the electron and proton Larmor frequency, respectively, and the correlation time τ_c is given by

$$\tau_c^{-1} = \tau_{\mathrm{R}}^{-1} + \tau_{\mathrm{M}}^{-1} + R_{1e} \tag{4}$$

The electron relation rate R_{1e} is described by the Bloembergen-Morgan equation (pseudorotation model)

$$R_{1e} = \frac{2\Delta_t^2}{50}[4S(S+1) - 3]\left(\frac{\tau_v}{1+\omega_S^2\tau_v^2} + \frac{4\tau_v}{1+4\omega_S^2\tau_v^2}\right) \tag{5}$$

where Δ_t^2 is the mean squared fluctuation of the zero-field splitting (ZFS), called squared transient ZFS, and τ_v is the correlation time for the instantaneous distortions of the metal coordination polyhedron.

Besides the overall molecular reorientation, occurring with correlation time τ_{R}, faster internal motions, with correlation time τ_{f}, can also modulate (at least partially) the electron-nucleus dipole-dipole interaction. In the Lipari-Szabo model-free treatment, Eq. (3) should thus be replaced by

$$R_{1M}=\frac{2}{15}\left(\frac{\mu_0}{4\pi}\right)^2\frac{\gamma_I^2 g_e^2\mu_B^2 S(S+1)}{r^6}\left[S_{\mathrm{LS}}^2\left(\frac{7\tau_c}{1+\omega_S^2\tau_c^2}+\frac{3\tau_c}{1+\omega_I^2\tau_c^2}\right)+(1-S_{\mathrm{LS}}^2)\left(\frac{7\tau_i}{1+\omega_S^2\tau_i^2}+\frac{3\tau_i}{1+\omega_I^2\tau_i^2}\right)\right] \quad (6)$$

where $\tau_i^{-1} = \tau_c^{-1} + \tau_{\mathrm{f}}^{-1}$.

The outer-sphere relaxivity is due to the water molecules diffusing around the paramagnetic metal ion, and, assuming that metal and ligand nuclei are in the center of hard sphere spherical molecules, according to one of the most commonly used models for diffusion

$$r_{1\mathrm{OS}}=\frac{32\pi}{405}\left(\frac{\mu_0}{4\pi}\right)^2\frac{N_{\mathrm{A}}\gamma_I^2 g_e^2\mu_B^2 S(S+1)}{aD}\left(7J^{\mathrm{tr}}(\omega_S)+3J^{\mathrm{tr}}(\omega_I)\right) \quad (7)$$

where N_{A} is the Avogadro constant, a is the distance of closest approach between the ligand nuclei and the paramagnetic metal ion, D is the sum of the diffusion coefficients of the paramagnetic molecule and of the ligand molecule, and

$$J^{\mathrm{tr}}(\omega)=\frac{1+\frac{5z}{8}+\frac{z^2}{8}}{1+z+\frac{z^2}{2}+\frac{z^3}{6}+\frac{4z^4}{81}+\frac{z^5}{81}+\frac{z^6}{648}} \quad (8)$$

with

$$z=\sqrt{2(\omega\tau_D+R_{1e}\tau_D)} \quad (9)$$

and

$$\tau_D=\frac{a^2}{D} \quad (10)$$

Zero-field splitting effects

Eqs. 3 and 6 are derived in the absence of static ZFS. In the presence of ZFS, the energies of the electron spin states can be very different from what predicted from the Zeeman term. These differences affect the transition probabilities between the different states, and thus the nuclear relaxivity. The extent of this effect largely depends on the magnitude of the static ZFS and on the position of the nucleus with respect to the *z* axis of the ZFS tensor. In the so-called slow rotation limit ($\tau_R \gg R_{1e}^{-1}$) and within the Redfield limit, the modified Florence NMRD program was used to calculate the relaxivity profiles as a function of these parameters (static ZFS and angle between the *z* axis of the ZFS tensor and the metal-water protons direction).

The effects of static ZFS on the relaxivity profiles become negligible at electron Larmor frequencies larger than the ZFS frequency (equal to ZFS in cm^{-1} times $2\pi c$, where c is the velocity of light). For Gd^{3+} complexes, where ZFS is smaller than 0.05 cm^{-1}, this corresponds to proton Larmor frequencies larger than ca. 10 MHz.

In vitro stability tests

In vitro stability tests were carried out on PEGylated nanogels in human serum (Seronorm™) at pH=7. To this purpose, the NG-2-PEG were diluted in human serum (Gd(III) concentration: 0.159 mM) and subjected to continuous gentle vortexing at 37°C. The R_1 was measured at 21.5 MHz and 25 °C at different time points, starting from time 0 to 170 hours post incubation (Figure S10). To investigate the potential release of the complex from the NG-2-PEG, two dialysis cycles (4 hours, 12 kDa) in HEPES buffer (NaCl 0.15 M, HEPES 3.8 mM, pH 7.3) were carried out soon after incubation at 37°C of the nanoparticles with either HEPES buffer (Gd(III) concentration 0.140 mM) or human serum. The residual Gd(III) concentration was measured after each dialysis cycle by the glass vial test (Figure S13).[1]

1) Rakhshan S, Alberti D, Stefania R, Bitonto V, Geninatti Crich S. LDL mediated delivery of Paclitaxel and MRI imaging probes for personalized medicine applications. *J Nanobiotechnology*. **2021**, *19*, 208.

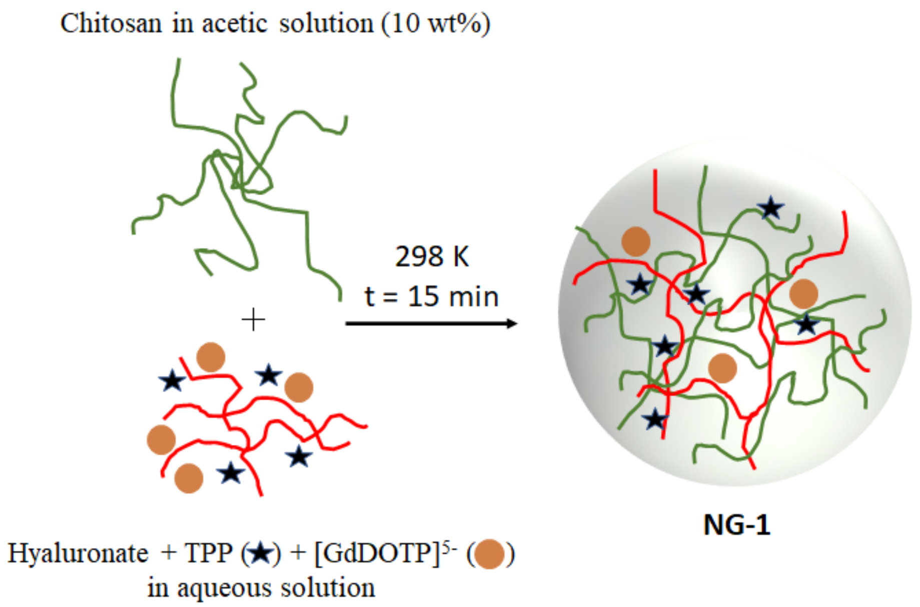

Scheme S1. Schematic view of the synthesis of NG-1 sample.

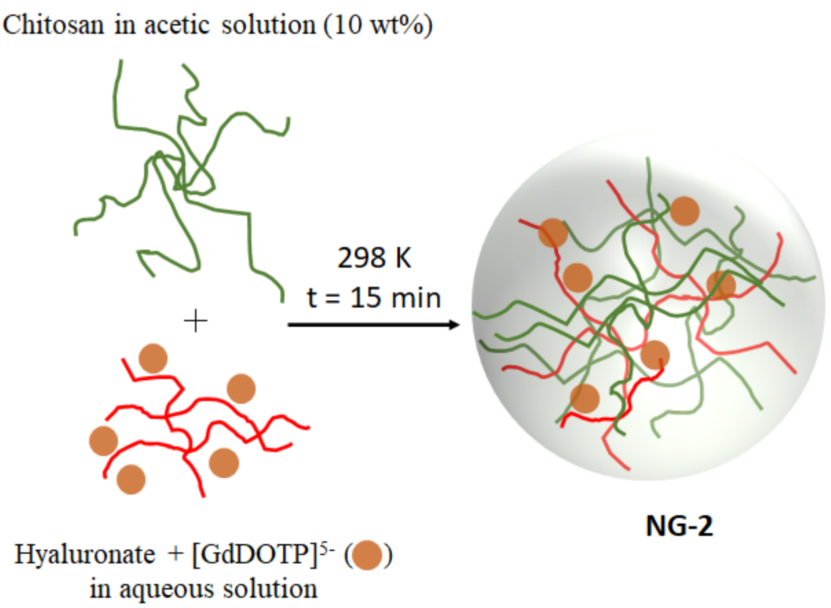

Scheme S2. Graphical illustration of the preparation of NG-2.

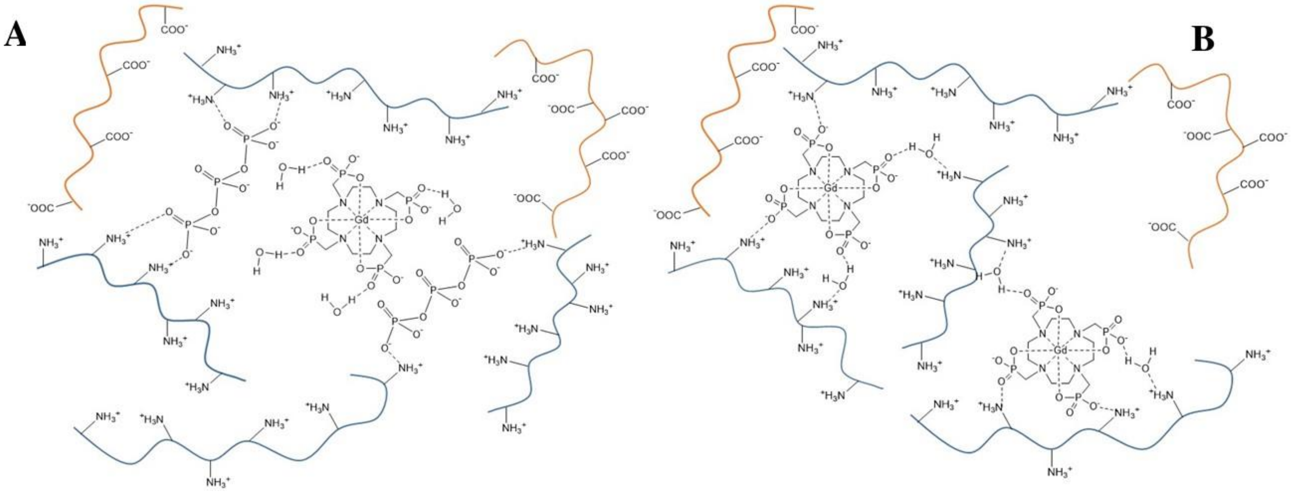

Figure S1. Schematic representation of the hydrogen-bonding network involving the second sphere water molecules of $[Gd(DOTP)]^{5-}$ in NG-1 (A) and NG-2 (B).

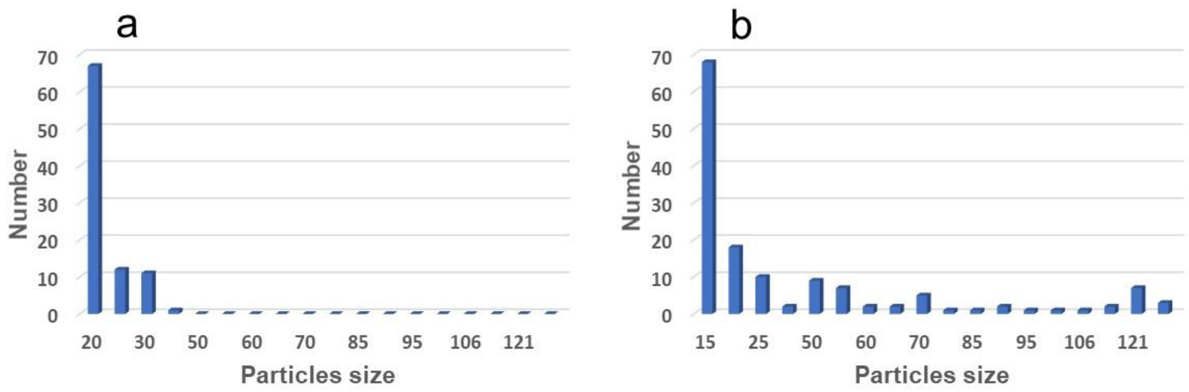

Figure S2. Particles size distribution for NG-1 (a) and NG-2 (b) nanogels (data obtained by counting about 100 particles).

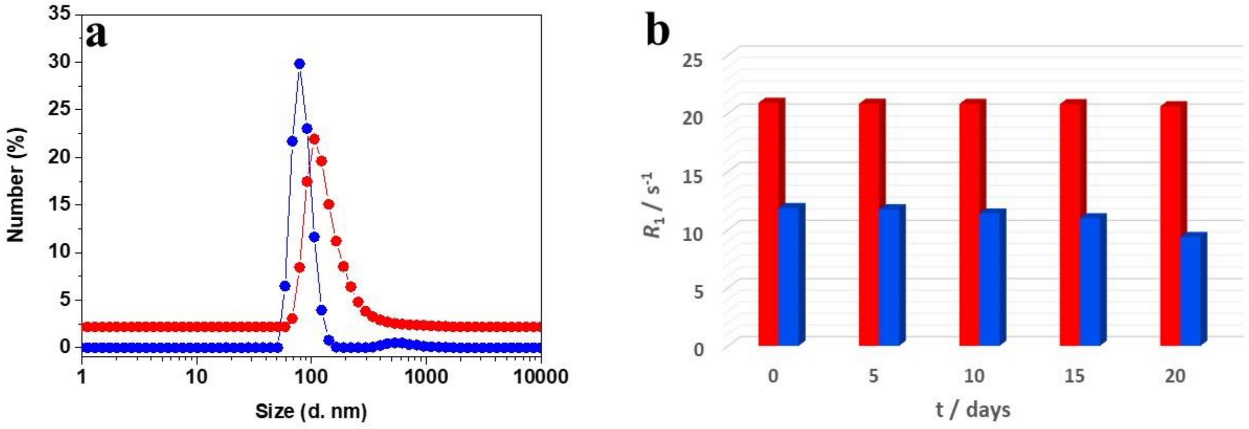

Figure S3. a) DLS analysis of NG-1 (•) and NG-2 (•) suspensions at 298 K and pH 5.4; b) time dependence of the longitudinal relaxation rate (R_1) at 10 MHz for NG-1 (blue) and NG-2 (red) suspensions at 298 K.

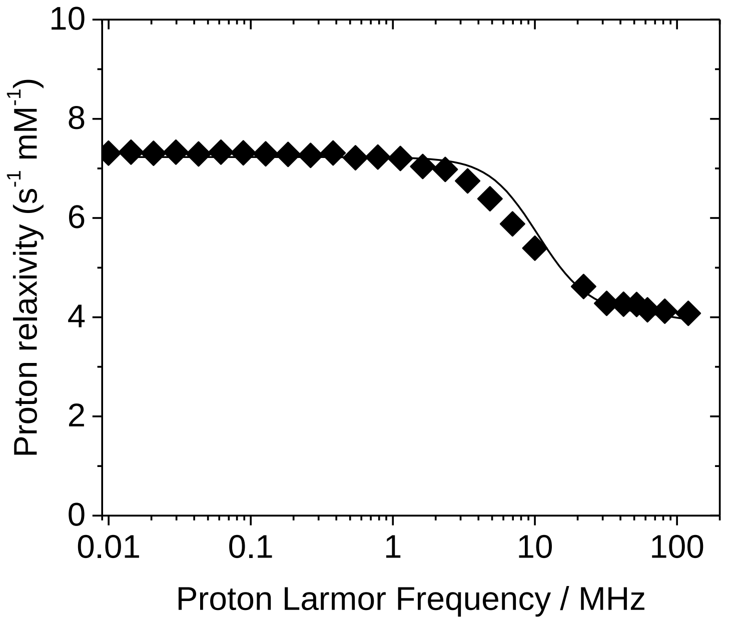

Figure S4. ^{1}H NMRD profile of $[GdDOTP]^{5-}$ at 298 K and neutral pH ($[Gd^{3+}]$ = 1.2 mM). The best-fit was obtained with the parameters of Table S1.

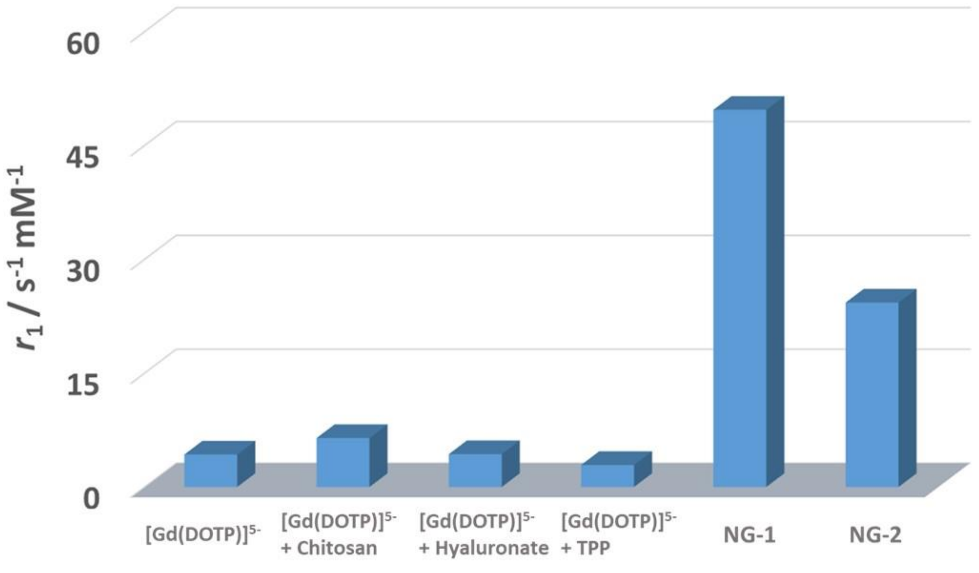

Figure S5. Relaxivity values at 298 K and 1.5 T of $[Gd(DOTP)]^{5-}$ (0.7 mM) in the presence of chitosan (1.6 mg/mL), sodium hyaluronate (0.3 mg/mL) and TPP (0.4 mg/mL) compared to the r_1 values of NG-1 and NG-1.

Table S1. Parameters from the analysis of ^{1}H NMRD data.

Parameters	$[GdDOTP]^{5-}$
Δ^2 (10^{19} s^{-2})	9.0±0.2
τ_V (ps)	17±1
τ_M (ns)	1.0[a]
τ_R (ps)	40±2
q^{SS}	4[a]
r^{SS} (Å)	3.5[a]
a (Å)	4.0[a]
^{298}D (10^5 cm^2 s^{-1})	2.24[a]

[a] parameters fixed during the analysis.

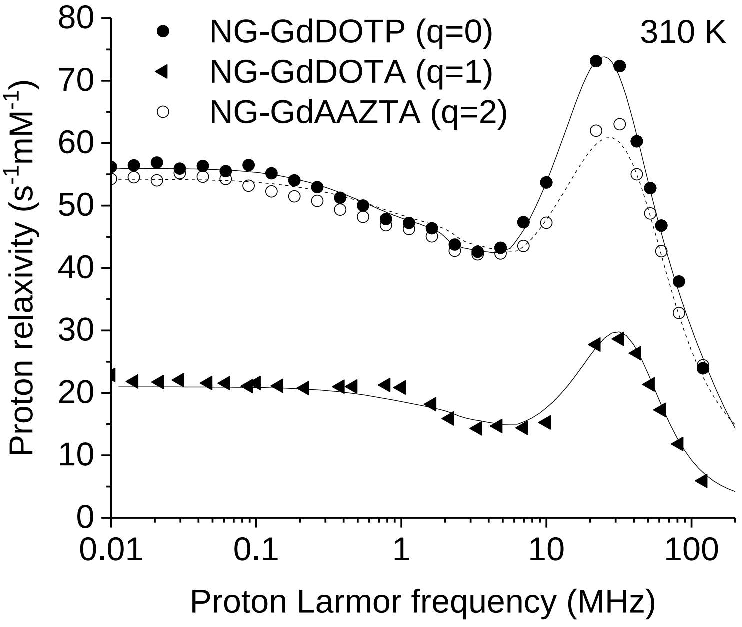

Figure S6. ^{1}H NMRD profiles of nanogel-embedded GdDOTP (NG-1, ([Gd^{3+}] = 0.18 mM)), GdDOTA ([Gd^{3+}] = 0.05 mM) and GdAAZTA ([Gd^{3+}] = 0.22 mM) at 310 K.

Table S2. Best fit parameters of the ^{1}H NMRD profiles shown in Fig. 3.

	**NG-1**			**NG-2**			
**T**	283	298	310	283	298	310	K
***r* (*)**	**3.5**			**3.5**			Å
q	3.1 ± 0.1			1.66 ± 0.08			
***a* (*)**	**4.0**			**4.0**			Å
***D* (*)**	**$1.5 \cdot 10^{-9}$**	**$2.3 \cdot 10^{-9}$**	**$3.0 \cdot 10^{-9}$**	**$1.5 \cdot 10^{-9}$**	**$2.3 \cdot 10^{-9}$**	**$3.0 \cdot 10^{-9}$**	m^2/s
Δ_t	0.012 ± 0.001			0.010 ± 0.001			cm^{-1}
τ_V	14 ± 2	14 ± 1	13 ± 2	19 ± 2	17 ± 1	15 ± 1	ps
τ_R **(*)**	≳ **100**			≳ **100**			**ns**
S^2	0.37 ± 0.08			0.34 ± 0.03			
τ_f	1.9 ± 0.5	1.5 ± 0.4	1.3 ± 0.3	1.4 ± 0.3	1.2 ± 0.2	1.0 ± 0.2	ns
τ_M	7 ± 2	6 ± 2	6 ± 2	7 ± 1	6 ± 1	5 ± 1	ns
**ZFS**	0.039 ± 0.004			0.032 ± 0.004			cm^{-1}
**angle**	55 ± 3			54 ± 3			°

(*) fixed.

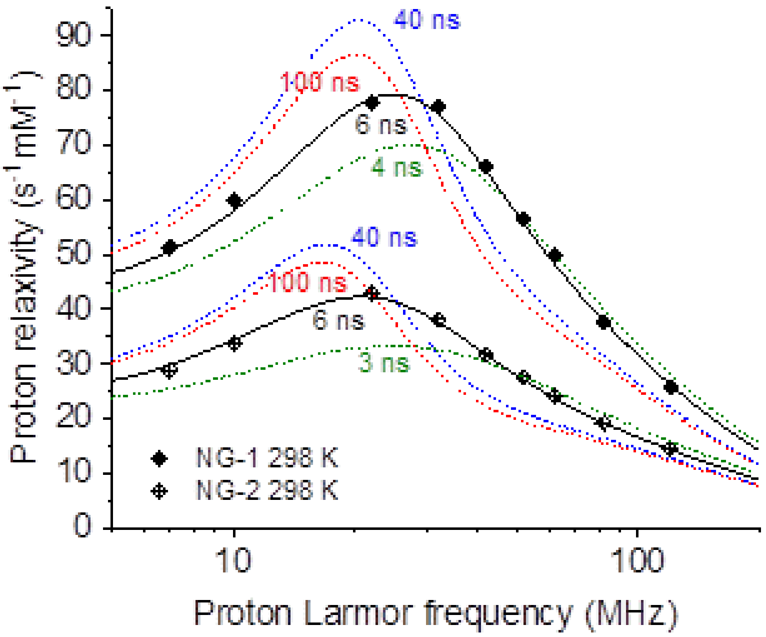

Figure S7. The experimental high field relaxivity of NG-1 and NG-2 at 298 K and the best fit profiles (black solid lines), obtained with τ_M = 6 ns, are shown together with theoretical relaxivity profiles (colored dotted lines) calculated for different τ_M values ranging from 3-4 to 100 ns. The relaxivity peak corresponds to τ_M of ca. 40 ns. Other parameters have the values reported in Table S2.

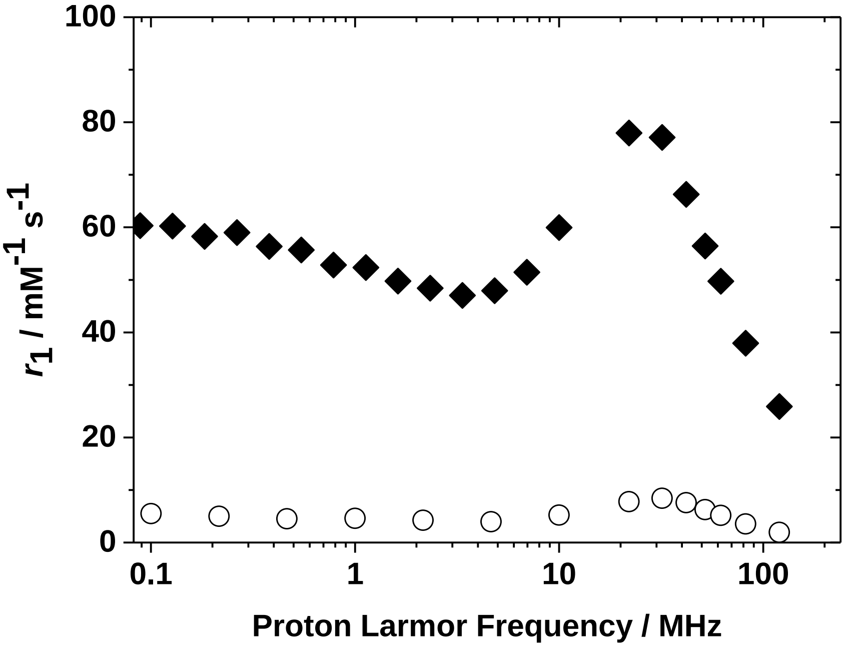

Figure S8. ^{1}H NMRD profiles of nanogel-embedded GdDOTP (NG-1 ([Gd^{3+}] = 0.18 mM), ♦) and Gd-TACN-PIC_3 (NG-3 ([Gd^{3+}] = 0.19 mM), ○) at 298 K.

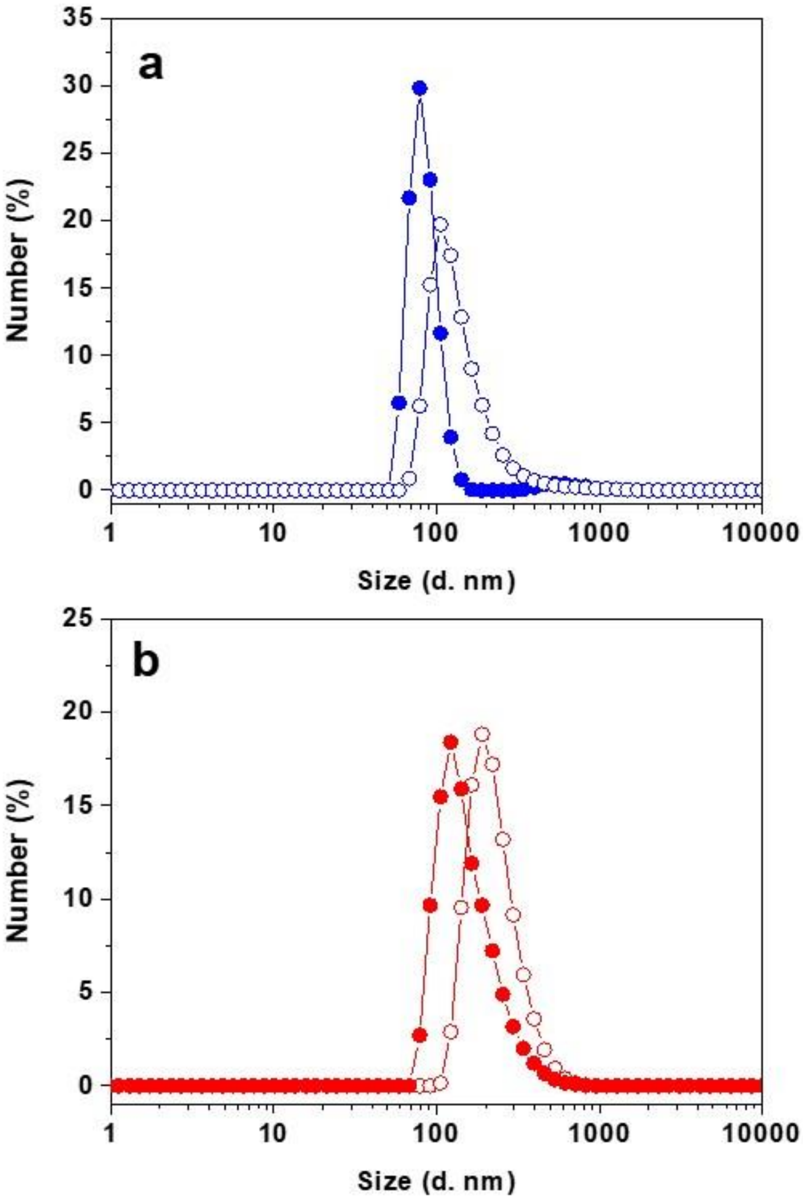

Figure S9. DLS analysis of a) NG-1 before (•) and after PEGylation (○) and b) NG-2 before (•) and after reaction with PEG (○) at 298 K.

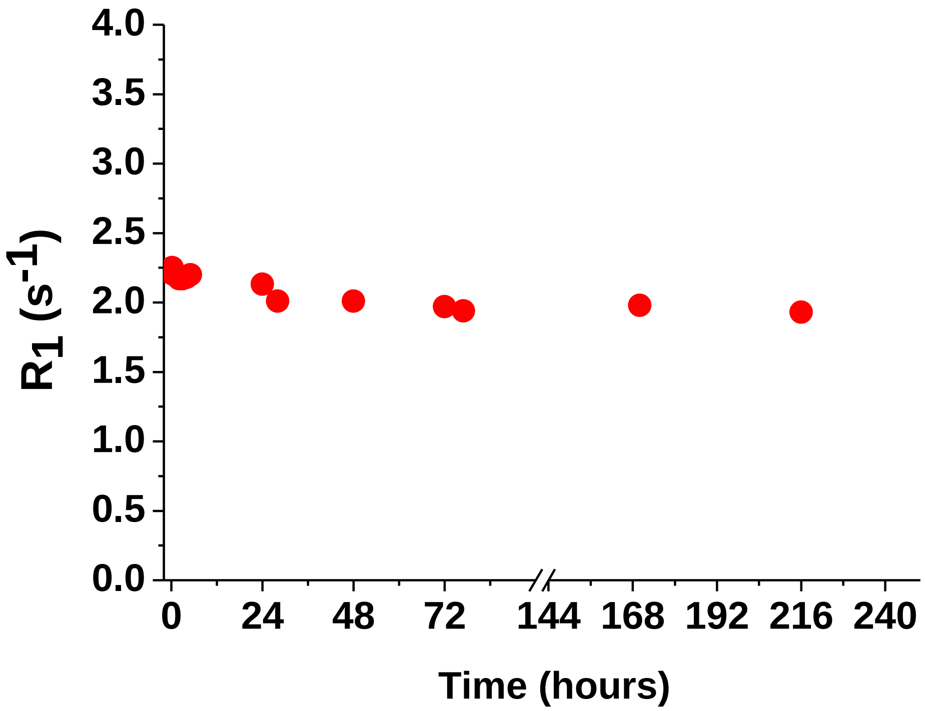

Figure S10. R_1 values of NG-2-PEG measured in human serum (Gd(III) concentration 0.159 mM) at 25°C, 21.5 MHz, at different times post incubation at 37°C under gentle vortexing.

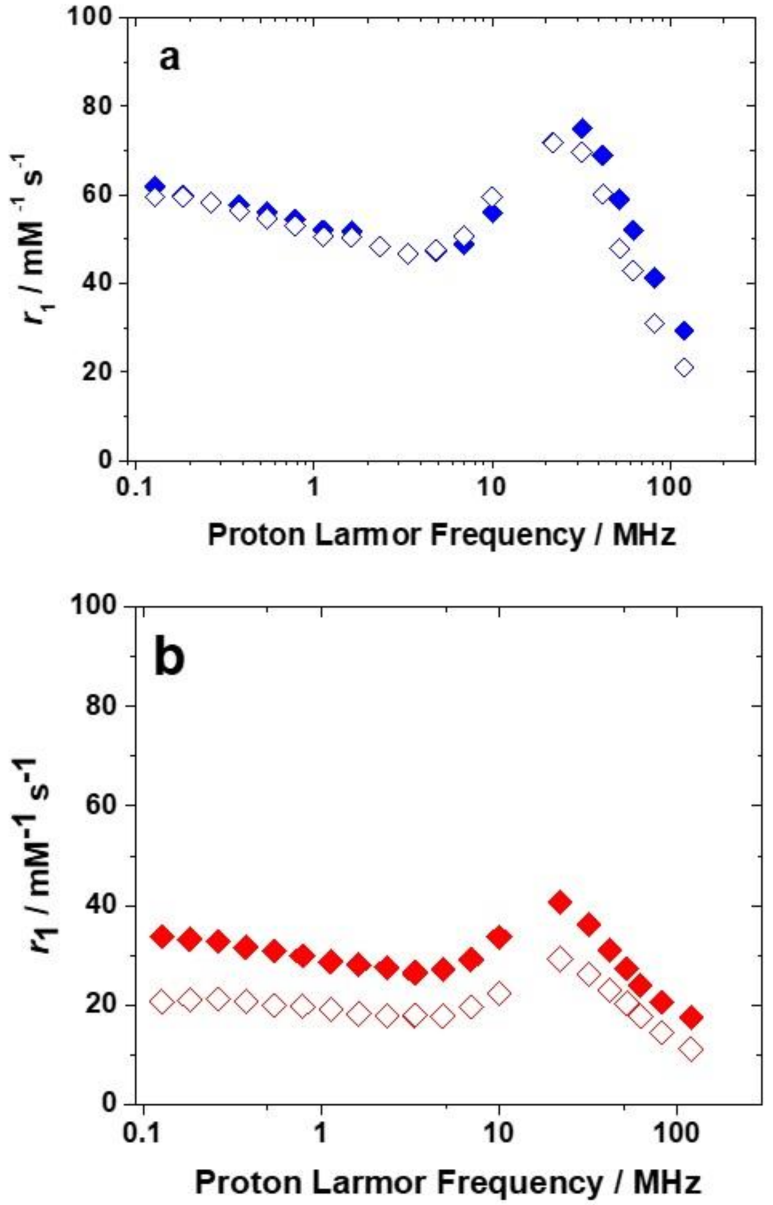

Figure S11. ^{1}H NMRD profiles at 298 K of: a) NG-1 before ([Gd^{3+}] = 0.18 mM) (♦) and after PEGylation ([Gd^{3+}] = 0.10 mM) (◊), b) NG-2 before ([Gd^{3+}] = 0.60 mM) (♦) and after PEGylation ([Gd^{3+}] = 0.34 mM) (◊).

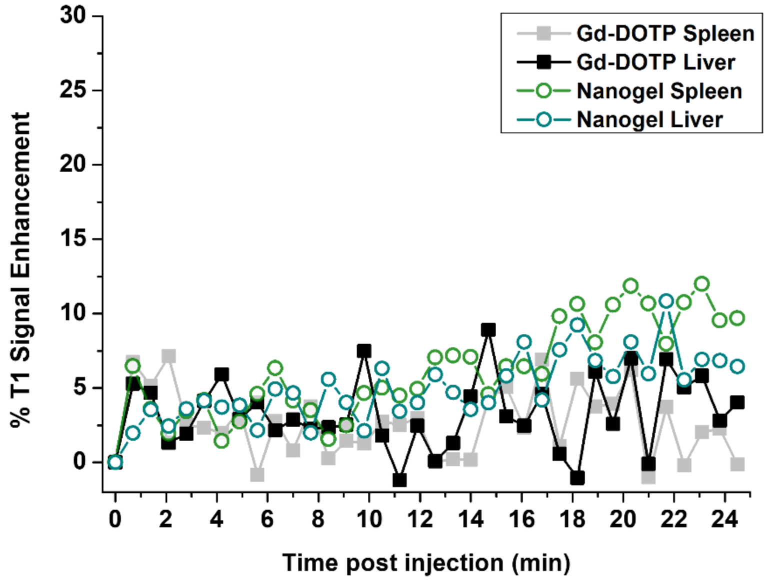

Figure S12. % T_1 signal enhancement over pre-images measured at 1T in the liver and spleen after the administration of the paramagnetic nanogel (empty circles) or $[GdDOTP]^{5-}$ (filled squares) (6.5 µmol Gd/Kg bw).

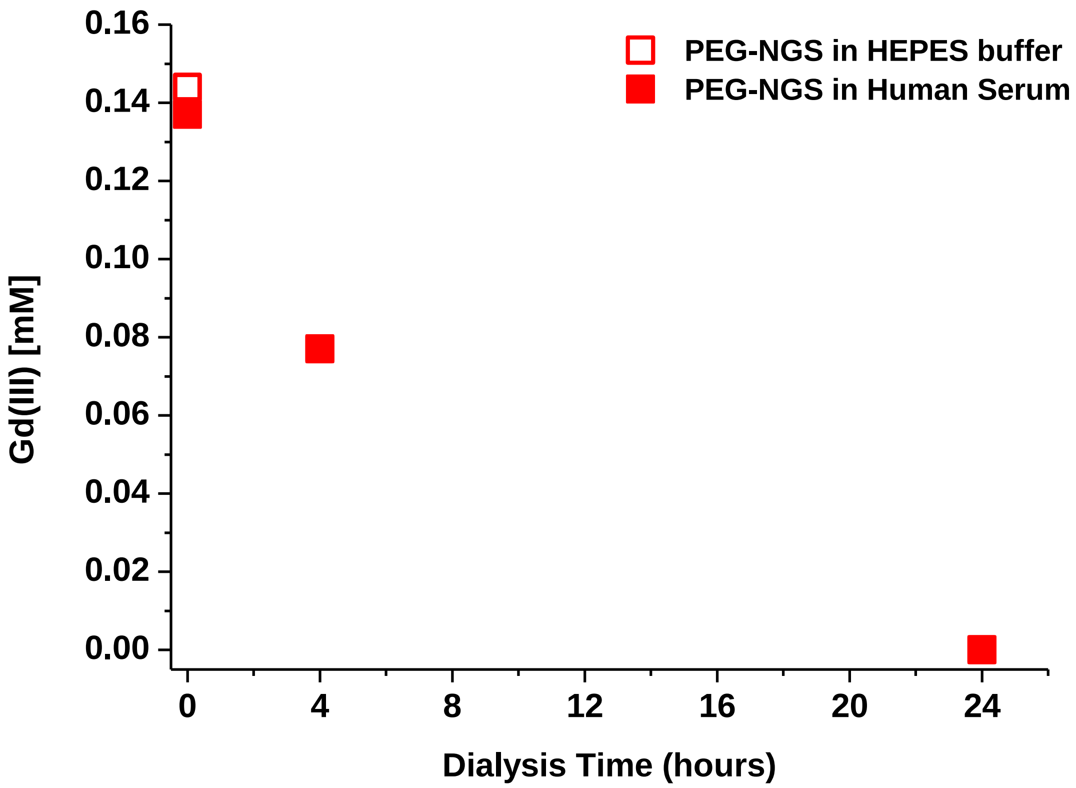

Figure S13. Residual Gd(III) concentration measured for NG-2-PEG suspended in either HEPES buffer (open squares) or human serum and subjected to dialysis (HEPES buffer, pH 7.3, 4°C, cut off: 12 kDa).
